# Effects of Process Parameters on the Fibrous Structure and Textural Properties of Calcium Caseinate Extrudates

**DOI:** 10.3390/polym15051292

**Published:** 2023-03-03

**Authors:** Ziqi Zhao, Zhaojun Wang, Zhiyong He, Maomao Zeng, Jie Chen

**Affiliations:** State Key Laboratory of Food Science and Technology, Jiangnan University, Wuxi 214122, China

**Keywords:** high-moisture extrusion, calcium caseinate, fibrous structure, textural properties

## Abstract

Textured calcium caseinate extrudates are considered promising candidates in producing fish substitutes. This study aimed to evaluate how the moisture content, extrusion temperature, screw speed, and cooling die unit temperature of the high-moisture extrusion process affect the structural and textural properties of calcium caseinate extrudates. With an increase in moisture content from 60% to 70%, there was a decrease in the cutting strength, hardness, and chewiness of the extrudate. Meanwhile, the fibrous degree increased considerably from 1.02 to 1.64. The hardness, springiness, and chewiness showed a downward trend with the rise in extrusion temperature from 50 °C to 90 °C, which contributed to the reduction in air bubbles in the extrudate. Screw speed showed a minor effect on fibrous structure and textural properties. A low temperature (30 °C) in all cooling die units led to damaged structure without mechanical anisotropy, which resulted from fast solidification. These results show that the fibrous structure and textural properties of calcium caseinate extrudates can be effectively manipulated by adjusting the moisture content, extrusion temperature, and cooling die unit temperature.

## 1. Introduction

Textured protein is commonly used as a raw material for meat analogs and is widely accepted by consumers owing to its good nutritional quality and environmental friendliness [1,2]. Various vegetable proteins, such as soy protein, pea protein, and wheat gluten, as well as some animal proteins, such as casein, whey protein, and insect protein, have been textured [3,4,5,6,7,8,9]. The extrusion temperature for textured vegetable protein (TVP) must be greater than 130 °C in order to melt the protein [10]. The high extrusion temperature used to produce conventional meat analogs triggers the Maillard reaction and caramelization which darken the color of the food [11,12]. This limits the application of TVP to red meat, or to less color-critical meat analogs, such as beef or pork analogs. However, it is not suitable as fish or shellfish substitutes, which need to be white.

To enhance the application of textured protein in fish substitutes or other color-critical food systems, white protein materials with low melting temperatures are preferred. Casein is the most abundant protein in milk and offers high nutritional value and bioavailability, which makes it a promising candidate [13]. Additionally, dairy products are ethically and morally acceptable to some vegetarians because the animals are raised for milk rather than slaughter. Furthermore, dairy products are a great protein source that meet nutritional needs. Calcium caseinate premixes can be processed using a shear cell to obtain the textured protein [14]. Caseins in calcium caseinate tend to self-assemble into caseinate micelles (100–300 nm) with attractive interactions between micelles due to the presence of Ca^2+^. These micelles can be aligned by shear force to form fibrous structures at the nanoscale [15]. The air incorporated in the calcium caseinate matrix acts as a weak phase perpendicular to the shear flow and enhances the material strength parallel to the shear flow. As a result, it promotes a fibrous appearance and mechanical anisotropy [16]. Shear cell devices can be used for batch processing and operate similarly to rheometers. Shear cell devices have been used successfully to produce several fibrous products with pea protein, soy protein, wheat gluten, and calcium caseinate, based on well-defined shear flow deformation and heating [17,18,19,20,21]. To date, this technology has been successful at the pilot scale [22].

Due to rapid developments in technique and equipment, extrusion is the dominant process used in the large-scale commercial production of TVP. In contrast to shear cells, high-moisture extrusion also involves shear flow inside the cooling die [23,24]. In addition, elongational (or extensional) flow occurs when the flow channel contracts inside the cooling die during food extrusion [25,26,27]. Thus, the biopolymers in the raw materials are subjected not only to shear and heat, but also to mixing, compression, and tension. High-moisture extrusion provides both shear and tensile stresses and is thus more effective and productive than a shear cell which only provides shear stress [28]. Clarifying the relationship between process parameters and product characteristics is important for increasing the applicability of high-moisture extrusion. Among the various parameters of the extrusion process, the extrusion temperature, moisture content, and screw speed significantly influence the properties of the extrudate [29,30,31]. In addition, the cooling die design is a key consideration for ensuring the formation of a fibrous structure and allowing new intra- and intermolecular bonds to form [32]. However, few studies have considered the effect of varying the cooling die design on textured proteins produced using high-moisture extrusion.

The objective of this study was to examine the effects of four process parameters, alone and coupled, on the extrudate characteristics from the high-moisture extrusion of calcium caseinate. The four process parameters were the extrusion temperature, moisture content, screw speed, and cooling die unit temperature. The cooling die unit temperature was varied by changing the combination of joinable units that were heated separately. The extrudate characteristics included textural properties, macro-structure, and microstructure. The rheological properties were also investigated. The characteristics of the extrudate were compared to those of cod (*Gadus morhua*), scallop (*Pectinidae*), and salmon (*Oncorhynchus mykiss*).

## 2. Materials and Methods

### 2.1. Materials

Calcium caseinate was purchased from Fonterra Casein (Edgecumbe, New Zealand). The calcium caseinate powder (dry-base) contained 91.1 wt% protein, 1.3 wt% fat, and 1.5 wt% calcium, according to the manufacturer’s specifications. The moisture content was 4.69 wt%. The aquatic products of cod (*Gadus morhua*), scallop (*Pectinidae*), and salmon (*Oncorhynchus mykiss*) were purchased from a local supermarket in Wuxi, China.

### 2.2. Preparation of Extrudates

**Figure 1:**
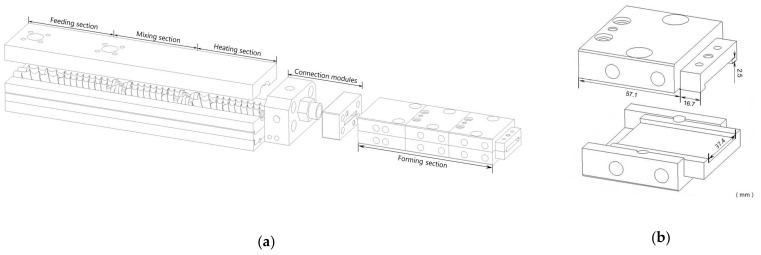
Schematic drawing of (**a**) the twin-screw extruder comprising a screw section (feeding section, mixing section, and heating section), connection modules, and a forming section; (**b**) the forming section composed of cooling die units.

Extrusion was performed using a co-rotating twin-screw extruder (ZE-16, 25:1 L/D, ATS, China) equipped with a screw diameter of 16 mm. The screw configuration is shown in Figure 1a. Three cooling die units were attached to the end of the connection modules (Figure 1b). Each unit had a separate water supply for temperature control. A spiral pipe cooling system was installed instead of a cooling jacket to increase the cooling efficiency.

The extrusion temperature was defined as the temperature of the heating section, which was the only section of the extruder barrel that was modified during experiments. The temperatures of the feeding section and mixing section of the extruder barrel were kept constant at 30 °C. Dry raw material was fed into the extruder at a consistent feed rate of 0.3 kg/h. A single-factor experiment was performed. For each of the four sets of trials, only one of the four variables was used: moisture content, extrusion temperature, screw speed, and temperature of cooling die units. These parameters are detailed in Table 1. The temperatures of the three cooling die units are shown along the direction of material flow. Three dead-stop trials were performed after the fourth set of trials. The extrudate samples were collected from the end of the cooling die after the process responses were constant for at least 5 min. The samples were then vacuum packed and stored at −20 °C until they were analyzed, with the exception of textural analyses (i.e., texture profile analysis and cutting strength analysis) which were performed on the samples within 1 h after discharge from the device.

### 2.3. Analysis of Textual Properties

#### 2.3.1. Texture Profile Analysis

The texture profile analysis (TPA) was performed using a TA.XT2 Texture Analyzer (Stable Micro-Systems, UK). Square specimens (10 mm × 10 mm × 5 mm) were cut from fresh extrudates and three aquatic products (i.e., cod, scallop, and salmon). Hardness, adhesiveness, springiness, cohesiveness, and chewiness were analyzed using the two-bite test as described by Chiang et al. [5], with modifications. Pieces were compressed using a P/36R probe to 50% of their original thickness at a speed of 1 mm/s for the first bite. They were then returned to their original position over 5 s, which was followed by the second bite at 1 mm/s to 50% of the first compressed thickness.

#### 2.3.2. Cutting Strength Analysis

The perpendicular strength, parallel strength, and fibrous degree of the extrudates and three aquatic products were analyzed as described by Osen et al. [33], with modifications. Pieces were cut using an A/CKB probe (knife blade) to 75% of their original thickness at a speed of 1 mm/s along the directions perpendicular (*F*_L_) and parallel (*F*_V_) to the outflow of the extrudate from the extruder. The fibrous degree was defined as the ratio of *F*_L_ to *F*_V_.

### 2.4. Macro- and Microstructures of the Extrudate

#### 2.4.1. Visual Analysis

Samples were torn along the direction of material flow to show thin isolated fibers once the extrusion process reached a steady state under a given condition. Each sample was then air-dried to become transparent. A digital camera (EF-S 18-135 mm f/3.5-5.6 IS USM, Canon 90D) was used to photograph the fibrous structure (shutter speed 1/160s, aperture f4.5, ISO 200), and a macro-lens (HMC-Nano-CPL, Soulmate, Beijing, China) was equipped to observe the morphology of air bubbles (shutter speed 1/400 s, aperture f5.6, ISO 1000). The three aquatic products were torn along the direction of the muscle and photographed in the same manner. The flow morphology of the extrudates was then analyzed. A 4 cm long section was cut from the extrudate strand and torn laterally in the flow direction. Charcoal powder was painted on the surface to make the cross-section easier to visualize.

#### 2.4.2. Scanning Electron Microscopy

The microstructure of the extrudates was characterized using SEM (SU8100, Hitachi High-Tech Co., Ltd., Tokyo, Japan). Samples were sliced into 5 mm × 5 mm × 2 mm (L × W × H) rectangles and fixed in glutaraldehyde solution (2.5 v%) for 12 h, followed with three rinses using 0.1 M phosphate buffer solution for 10 min. Then, a gradient of 30%, 50%, 70%, 90%, and 100% (v%) ethanol solutions were used to dehydrate each sample for 10 min. The samples were then placed in a fume hood overnight to evaporate residual ethanol, then dried using a vacuum freeze drier, attached onto holders, and sputter-coated with 10-nm-thick layers of wolfram. They were then viewed using SEM at an accelerating voltage of 3.0 kV at 100× magnification.

#### 2.4.3. Light Microscopy

The muscles of the three aquatic products were cut using a knife into small blocks (5 × 5 × 2 mm^3^) in the direction of the muscle. They were then fixed in Bouin solution (70% saturated picric acid, 25% formaldehyde, and 5% acetic acid) at 4 °C for 24 h. The samples were then dehydrated at 4 °C with a graded series of ethanol solutions (70–90% in 5% increments) followed with incubation in ethanol–xylene (1:1) for 1 h and then xylene for 1 h. The muscle samples were embedded in paraffin wax and cut longitudinally to the muscle fiber into 5 µm thick slices to prepare frozen sections. The sections were deparaffinized with toluene and rehydrated. These samples were stained with Masson’s trichrome as described by Alizadeh et al. [34], where the myofibers were stained red and the connective tissues were stained blue. The slides were observed using a light microscope at 100× magnification (DM 2000, Leica Microsystems CMS GmbH).

### 2.5. Temperature Sweep

Small-amplitude oscillatory strain (SAOS) experiments were performed with a temperature sweep to determine the thermo-rheological properties of the calcium caseinate premixes at moisture contents of 60%, 65%, and 70%. Measurements were carried out using a DHR-3 rheometer (TA Instruments, New Castle, DE, USA) with parallel plate geometry (diameter of 40 mm, gap of 1 mm). The circular samples were transferred from the premixes to the rheometer. The edges were covered with silicon oil to prevent desiccation. First, the temperature was increased from 30 °C to 90 °C at a linear rate of 5 °C/min. Then, the samples were cooled down to 30 °C at the same linear rate. The amplitude was a constant strain of 1%, and the frequency was set to f = 1 Hz (i.e., linear viscoelastic region of the calcium caseinate premix). The storage modulus (G′), loss modulus (G″), complex viscosity (η*), and loss tangent (tan δ = G″/G′) were recorded as functions of time. The gel–sol transition temperature (*T*_gel–sol_) was defined as the temperature at which the storage modulus and loss modulus were equal (i.e., G′ = G″; tan δ = 1).

### 2.6. Statistical Analysis

Samples were analyzed in triplicate. Analysis of variance (ANOVA) was performed to study the effects of each treatment on the textural properties of the extrudate. The least significant difference test (*p* ≤ 0.05) was applied to establish the differences between the means. The program Statistix version 9.0 (Analytical Software, Tallahassee, FL, USA) was used for the statistical analysis.

## 3. Results and Discussion

### 3.1. Effects of the Extrusion Parameters on the Textural Properties of the Extrudates

**Figure 2:**
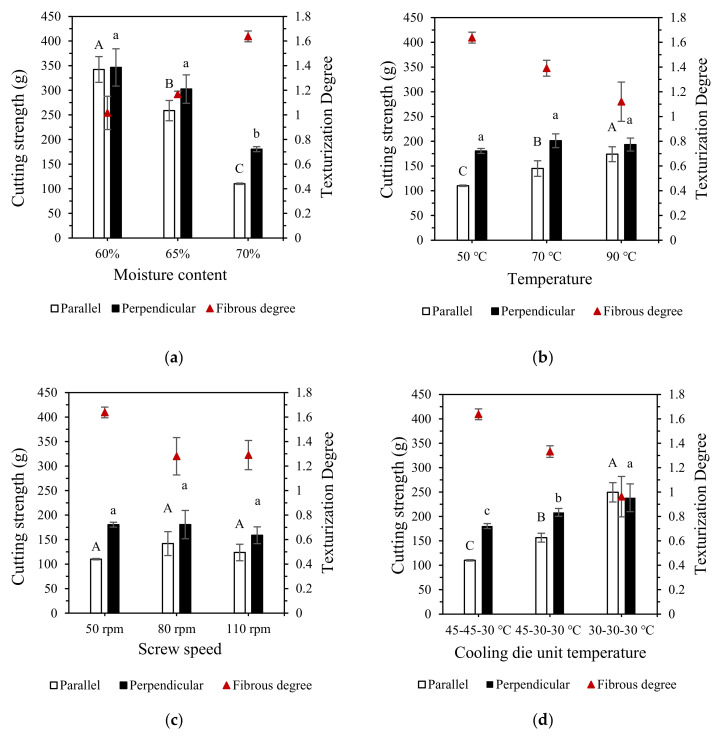

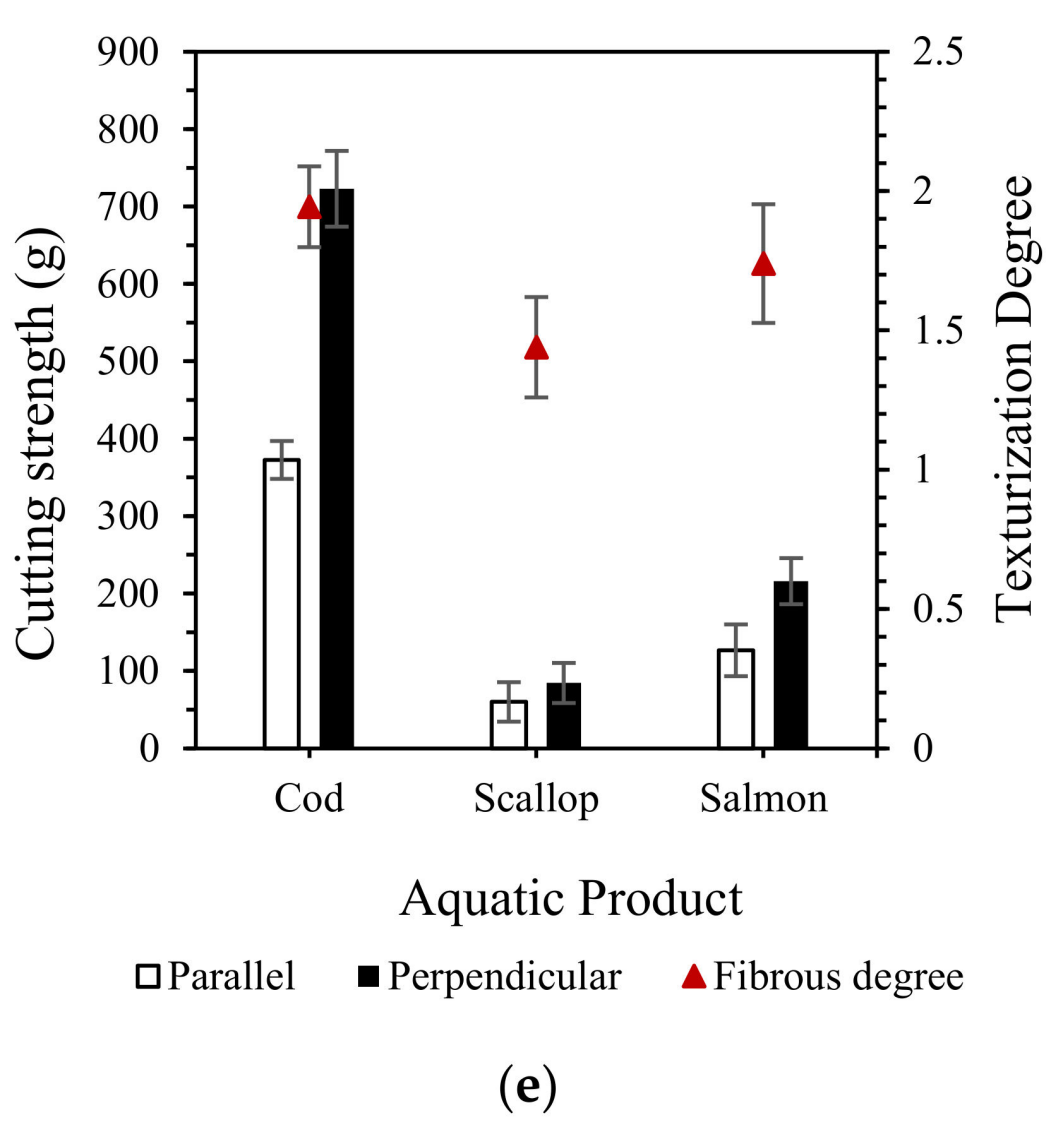
Cutting strength and texturization degree of the extrudates under different processing conditions as well as the three aquatic products: (**a**) moisture content, (**b**) extrusion temperature, (**c**) screw speed, (**d**) cooling die unit temperature and (**e**) aquatic product. Different letters indicate significant differences (Least Significant Difference test, *p* < 0.05) between mean values (±SD, *n* = 3).

The perpendicular strength, parallel strength and fibrous degree of the extrudate under different extrusion conditions are shown in Figure 2. The TPA results of extrudates are shown in Table 2. Increasing moisture content significantly decreased the perpendicular strength and parallel strength and significantly increased the fibrous degree (Figure 2a). At 60% moisture content, the fibrous degree dropped to approximately 1.02. As a type of plasticizer, moisture content has a significant impact on viscosity [35,36]. In this study, the increase in moisture content caused a reduction in the hardness, cohesiveness, and chewiness of extrudates, while increasing its adhesiveness (Table 2). A higher moisture content can increase the flexibility and mobility of protein molecules, which increases the potential for the reactive sites of calcium caseinate to come within sufficient proximity to facilitate cross-linking [23,37]. Decreasing the moisture content increases the viscosity, torque, and specific mechanical energy (SME), which inhibits cross-linking [38]. Moreover, excess water causes proteins to aggregate less, denature less, and have a looser structure [39]. The effects of the moisture content on the textural properties of calcium caseinate extrudate are similar to those of conventional vegetable protein extrudate, but the effects on the fibrous structure are different. The hardness, chewiness, and fibrous degree of the extrudate decrease with increasing moisture content for vegetable proteins such as soy, pea, and lupin [30,31,40,41,42]. The differences in the effects of the moisture content on the fibrous structures of the calcium caseinate and vegetable protein extrudates may be related to differences in the protein denaturation temperature, flow behavior, flexibility of protein molecules, and formation mechanism of the fibrous structure during extrusion [43].

The parallel strength of the extrudate increased significantly with increasing temperature, while the fibrous degree of the extrudate had a clear tendency to decrease, and the perpendicular strength remained roughly unchanged (Figure 2b). The extrusion temperature showed a significant positive correlation with cohesiveness and a negative correlation with hardness, springiness, and chewiness (Table 2). Extrusion temperature is an important factor when ensuring the material melts and for the subsequent organization of the extrudate [29,44]. The extrusion temperature affects the extrudate structure differently, depending on the protein type as well as the moisture content. The study of high-moisture extrusion of pea protein and lupin protein, for example, showed that the effects of extrusion temperature on cutting strength and hardness of the extrudates are influenced by moisture. The results show that cutting strength and hardness of the extrudate increases with extrusion temperature at low moisture (55%). At a high-moisture content (70%), increasing the extrusion temperature has little effect on cutting strength, or causes a slight decrease [33,41]. While Lin et al. [40] found that an increase in extrusion temperature significantly decreases the hardness and chewiness of extrudates at low moisture levels.

We found that increasing the screw speed decreased the fibrous degree (Figure 2c). Decreasing the screw speed significantly decreased hardness and chewiness. The screw speed had a negligible effect on springiness and cohesiveness (Table 2). A higher screw speed increased the SME and the flow rate of the material. Meanwhile, a lower screw speed allowed for longer residence time at the same temperature, which hydrated the material more fully when heated [45]. During high-moisture extrusion of vegetable proteins, the screw section is considered a bioreactor that applies thermal and mechanical stress for texturization [46]. However, for the high-moisture extrusion of calcium caseinate, the screw section primarily serves as a propeller rather than a reactor. The formation of textured calcium caseinate does not require high mechanical stress to unfold the protein structure, only a suitable shear flow. Excessive shear would instead damage the already-formed fibrous structure [15,31,43]. Therefore, the number of kneading blocks in the screw section was reduced.

Decreasing the cooling die unit temperature gradually decreased the fibrous degree to less than one (Figure 2d). Decreasing the cooling die unit temperature had similar effects on textural properties as increasing the screw speed, which may be related to higher SME (Table 2). Considering the four process parameters of this experiment comprehensively, the setting of the cooling die unit temperature seemed to be the most important, as it determined whether the fibrous structure that parallel to the direction of flow could be obtained. During the cooling process, the protein-rich and water-rich domains in the melt elongate at particular shear and temperature gradients. The elongated domains then solidify and form the fibrous structure with the further reduction in the melt’s temperature [47]. The temperature of the cooling die determines the pressure, shear stress, tensile stress, and melt flow state of the extrudate, resulting in different fibrous structures [43,48]. In an experiment of the extrusion texturization of renneted casein-based gels, the fibrous degree of the casein-based extrudates varied from 1.9 to 4.1, and the shear stress varied from 1.8 kPa to 44.1 kPa at different cooling die temperatures [6].

When the extrudate was compared to the aquatic products, the cod and extrudate had similar hardness at 60% moisture content, and the salmon and extrudate had similar hardness at 65–70% moisture content (Table 2). All three aquatic products had lower springiness than the extrudate. The cod and extrudate had similar chewiness at 70% moisture content. The cutting strengths of the cod in the perpendicular and parallel directions were up to 700 g and 300 g, respectively (Figure 2e). It also had a high fibrous degree of 1.94. The fibrous degree and cutting strength of the scallop were slightly lower than that of the extrudate at 70% moisture content. The fibrous degree and cutting strength of the salmon were close to those of the extrudate at 70% moisture content.

### 3.2. Structure of the Extrudates

**Figure 3:**
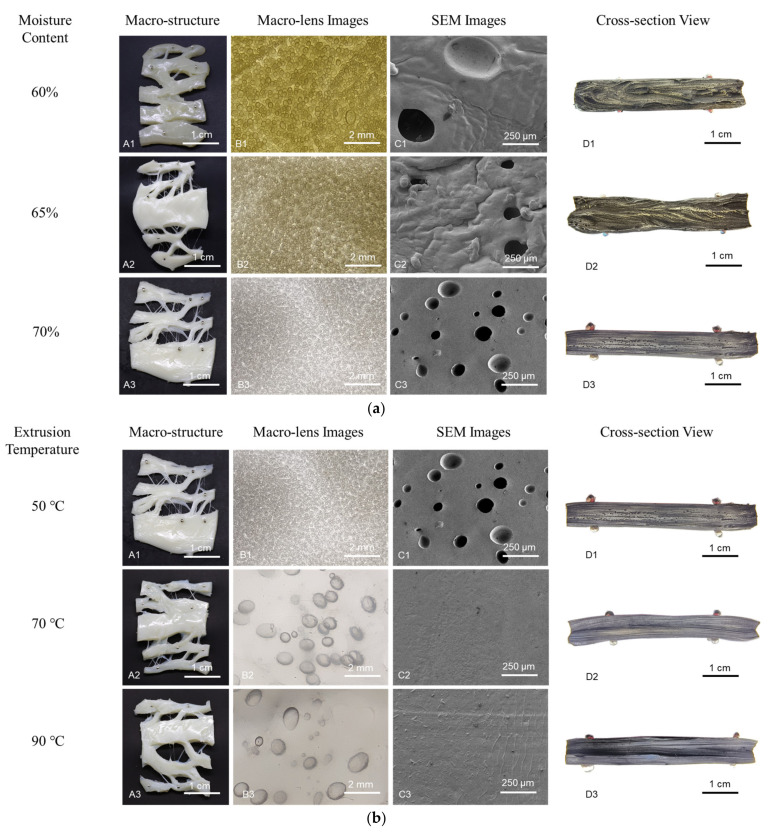

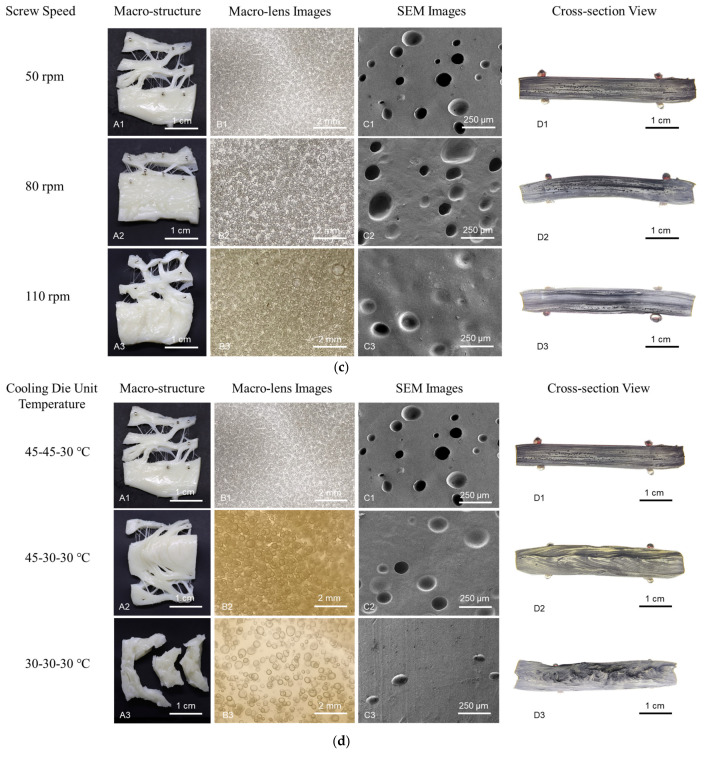

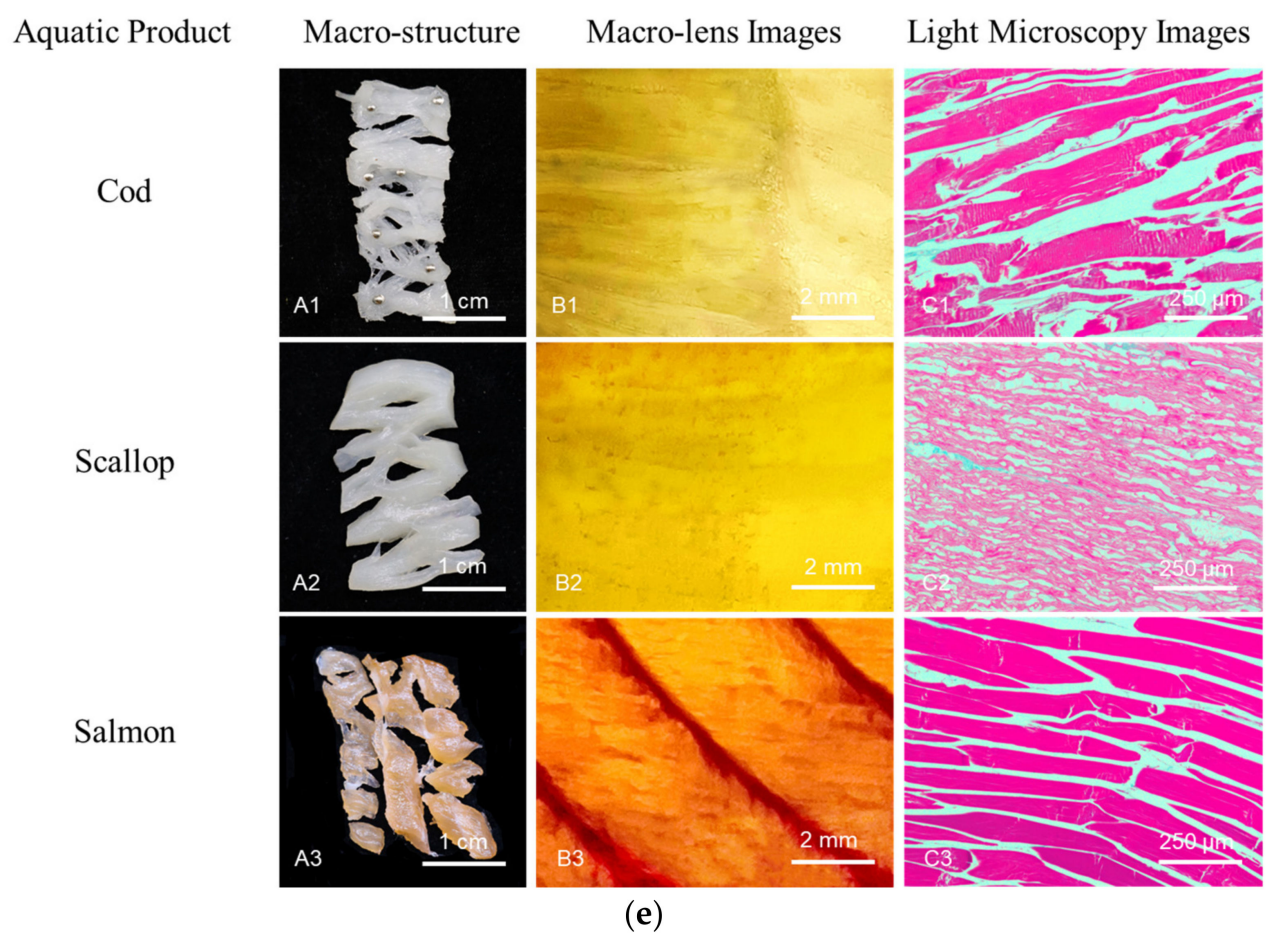
Macro-structure images, macro-lens images, SEM images, and cross-section views of samples under different test conditions: (**a**) moisture content, (**b**) extrusion temperature, (**c**) screw speed and (**d**) cooling die unit temperature; (**e**) macro-structure images, macro-lens images, and light microscopy images of three aquatic products. The flow direction is from right to left. The muscle fibers are in the horizontal direction.

Figure 3 shows macro-structure, macro-lens images, SEM images, and cross-section images of the calcium caseinate extrudates under different experimental conditions. The SEM images show the morphology of air bubbles inside the extrudate. A digital camera equipped with a macro-lens was used to corroborate observed trends over a wider view.

In the moisture content of the 60~70% range, the extrudates showed smaller fibers and a whiter appearance with increasing moisture content (Figure 3a(A1–A3)). At 60% moisture content, the extrudate was transparent and had an anisotropic structure upon tearing, but lacked tiny fibers (Figure 3a(A1)). Decreasing the moisture content reduced the number of bubbles and increased the bubble size (Figure 3a(B1–B3,C1–C3)). This may be because a lower moisture content allows the premixed material to form larger clumps, which leads to larger voids in the melt. The cross-section view shows a V-shape morphology, elongated in the flow direction and becoming parallel when the moisture content was increased (Figure 3a(D1–D3)). The V-shape at 60% moisture content indicates flow instability, which is in line with the results shown in Figure 2a. With increasing moisture content, the fibrous degree of extrudates was higher and the fibrous structure was more parallel.

Macro-structure images suggest that increasing the extrusion temperature (70 °C and 90 °C) decreased the number of tiny fibers in the extrudate (Figure 3b(A1–A3)). Among the process parameters considered in this study, the extrusion temperature had the most significant influence on the morphology and the number of bubbles. Increasing the extrusion temperature decreased the number of bubbles and increased the bubble size (Figure 3b(B1–B3)), which suggests that the extrusion temperature greatly influences the fibrous degree of the extrudate owing to its effect on the air bubbles (Figure 2b). SEM images showed no air bubbles at 70 °C and 90 °C, which can be attributed to the limited area selected (Figure 3b(C1–C3)). According to Wang et al. [16], the extrudate breaks along air bubbles when torn along the shear flow direction. The bubbles make the material less susceptible to failure in the parallel direction and more susceptible to failure in the perpendicular direction, which promotes anisotropy. The tiny fibers in the extrudate decrease upon tearing, as the number of bubbles decrease and the bubble size increases. A structure without bubbles breaks into large pieces upon tearing. In addition, intensive thermal treatment can also cause protein aggregation in the form of larger particle sizes, which is detrimental to the formation of the fibrous structure [14]. As can be seen from the clearer flow morphology, materials with higher temperatures have better flowability (Figure 3b(D1–D3)). This may be linked to the effect of temperature on the viscosity of materials.

Decreasing the screw speed resulted in an extrudate with a smoother appearance (Figure 3c(A1–A3)). The screw speed had little influence on the air bubble morphology and the flow morphology in the extrudate (Figure 3c(B1–B3,C1–C3,D1–D3)). These results align with the textural properties of the extrudate (Figure 2c and Table 2). As the screw speed increases, the depth of the pit in the SEM images is clearly shallow. It implies that the volume of bubbles slightly increases with increasing screw speed (Figure 3c(C1–C3)). A shear thinning flow behavior of calcium caseinate premixes may explain this [49]. Decreasing the cooling die unit temperature increased the cooling speed, which caused the fibrous appearance to disappear (Figure 3d(A1–A3)). When all three units were set to 30 °C, the extrudate showed a damaged structure (Figure 3d(A3)). The number of bubbles in the extrudate decreased with increasing cooling speed (Figure 3d(B1–B3,C1–C3)). These results may be explained by the quick solidification of the material, while the additional deformation in the cooling die damaged the structure [21].

**Figure A1:**
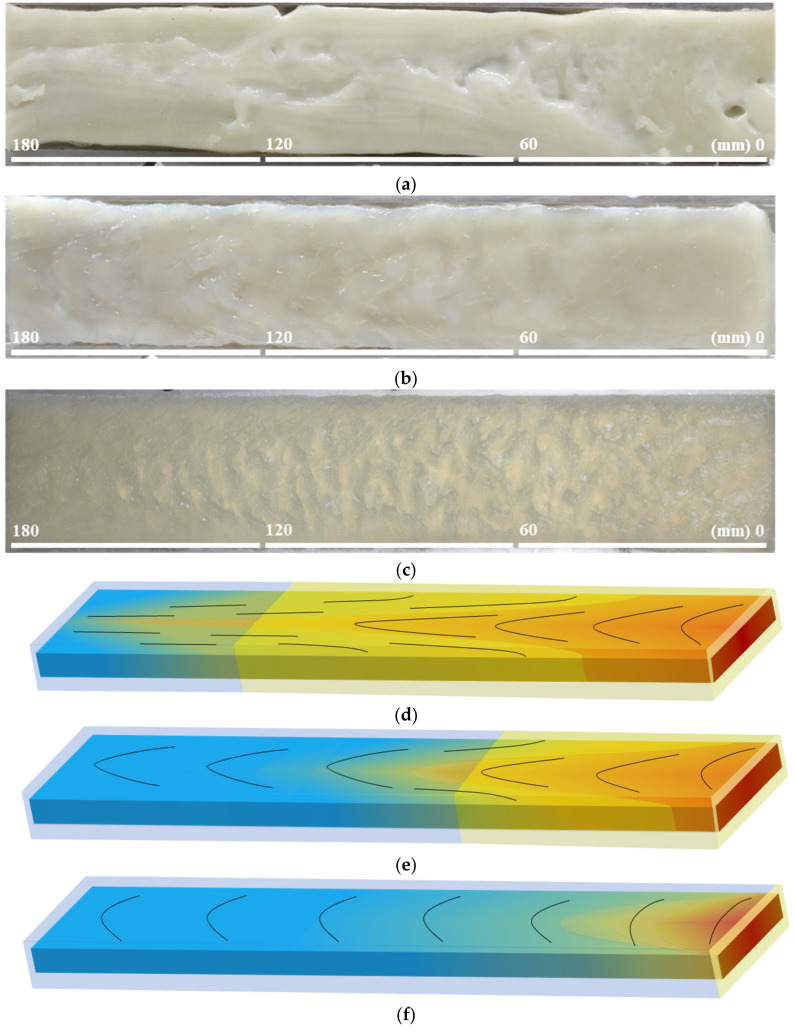
Structural appearance of viscoelastic material in the forming section after the dead-stop trials (**a**–**c**) and example illustration of the temperature profile with predominant flow pattern (black lines) under the corresponding conditions (**d**–**f**). The white bar represents the distance traveled by materials in the sheet die. The flow direction is from right to left.

The flow pattern and fiber alignment along the cooling die were studied and visualized with dead-stop trials after the fourth set of trials (i.e., at different cooling die unit temperatures). The temperature profile and predominant flow pattern are emphasized by black lines in the example illustrations (Figure A1d–f) based on physical images (Figure A1a–c). The plasticized material (50 °C) entered the forming section with a Hagen–Poiseuille flow profile owing to friction against the wall. In the forming section with a cooling unit (30 °C) only, the solidification of the material arrested the created anisotropic structure rapidly approximately 30 mm in the section, and the front Hagen–Poiseuille flow profile was retained until extrusion (Figure A1c,f). Under this condition, the plug flow took up most of the space in the cooling die, and the fibrous structure was oriented nearly transversely to the flow direction. For the other two kinds of cooling die temperature settings which had warming units (45 °C), the material closed to the wall solidified first and formed a contracted flow channel for the plasticized material at the core (Figure A1a,b,d,e). This generated an elongational flow. The first sample was extruded with two warming units (cooling die unit temperature: 45-45-30 °C) (Figure A1a,d). Elongated casein-based anisotropic structures gradually formed and aligned in the flow direction until the flow channel contracted to nothing. Finally, the material solidified further in the cooling section, which resulted in a parallel plug flow until extrusion. With one warming unit, the plasticized material solidified before the parallel structure formed (Figure A1b,e). The same trends were observed in the corresponding cross-section views (Figure 3d(D1–D3)). When the material at the front of the cooling section cooled into a solid state, it could not flow to form a velocity gradient, and blocked the viscoelastic material behind it. A continuum mechanics simulation showed that the solidification of the material could cause a plug flow that limited the amount of shear strain and arrest the created fibrous structure [47]. A dead-stop experiment proved that a more pronounced V-shape morphology developed in the transition section and non-cooling section, compared to that in the cooling section [28].

The shear and tensile stress inside the cooling die guide interactions between calcium caseinate micelles to extend along the flow direction. A simple shear flow contains a significant rotational component, which allows long molecules to rotate in the flow rather than deform. This is not sufficient for caseinate micelle chains to align. In contrast, an elongational flow has a deformation component greater than its rotational component, so it is the most efficient “chain stretcher” [50]. When a polymer solution or melt is stretched by an extensional flow, the molecules align more effectively in the stretching direction [51]. Changing the extrusion process parameters can influence the flow morphology to different extents. Materials with good flowability under particular processing conditions can form elongational and shear flows, which then form fibrous structures.

The macro-structure of the scallop was similar to that of the extrudate at 60% moisture content, and the macro-structure of the cod was similar to that of the extrudate at 65% and 70% moisture contents (Figure 3e(A1,A2)). The presence of connective or adipose tissues can be seen in the macro-lens images. They prevent the formation of long muscle tissues in cod and salmon, which also have no air bubbles (Figure 3e(B1–B3)). The micro-structures of the aquatic products were further observed with light microscopy (Figure 3e(C1–C3)). In comparison, the extrudate does not show a fibrous structure at the micro-scale according to the SEM images, only bubbles that deform in the flow direction. Thus, although the calcium caseinate extrudate is similar to the aquatic products in terms of the macro-structure and textural properties, the microstructure is quite different. The extrudate has too many air bubbles and lacks a microstructure. This may help explain why meat analogs produced with extrusion are consistently less flavorful than real meat.

### 3.3. Thermo-Rheological Properties

**Figure 4:**
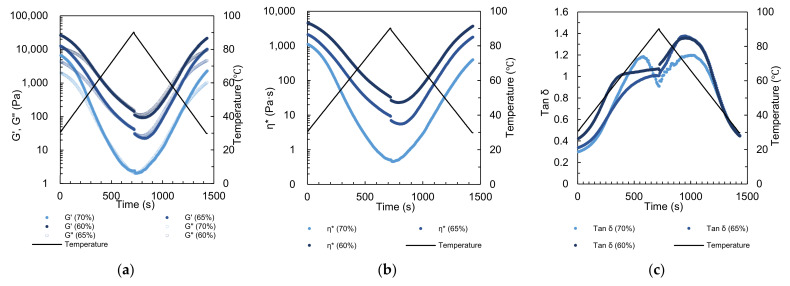
Calcium caseinate premixes at moisture contents of 60%, 65%, and 70% during temperature sweep experiments: (**a**) storage modulus (G′) and loss modulus (G″), (**b**) complex viscosity (η*), and (**c**) loss factor (tan δ).

To investigate the effects of different extrusion conditions on the fibrous and textural properties of the calcium caseinate extrudate, SAOS experiments with a temperature sweep were performed to measure the rheological properties during the heating and cooling processes. The storage modulus (G′) and loss modulus (G″) of the calcium caseinate premixes decreased with increasing moisture content and extrusion temperature (Figure 4a). The complex viscosity of the premixes showed the same trend as G′ and G″, which indicates that the flowability changed with the temperature (Figure 4b).

The gel–sol transition temperature (*T*_gel–sol_) during temperature-rise and temperature-fall periods differed depending on the moisture content (Table 3). During the temperature-rise period, *T*_gel–sol_ increased from 61.3 °C to 83.4 °C when the moisture content increased from 60% to 65%, and decreased to 66.7 °C when the moisture content increased further to 70%. During the temperature-fall period, *T*_gel–sol_ was approximately 50 °C. This may be because increasing the moisture helps dissolve calcium ions in the premix, which promotes calcium cross-linking [52,53]. Increasing the calcium ion concentration leads to phase separation because calcium ions facilitate the formation of a dense domain [54]. Pitkowski et al. [55] found that increasing the concentration of calcium ions decreases the solubility of calcium caseinate in aqueous solutions. Therefore, the moisture content affects the calcium ion content of hydrated calcium caseinate and the soluble-to-insoluble calcium caseinate ratio. The *T*_gel–sol_ of the premix is then affected. Calcium crosslinks between caseins are the most dominant bonds within calcium caseinate. When calcium-mediated casein–casein interactions are strong, more thermal energy is required to achieve the gel–sol transition, which is reflected in the higher *T*_gel–sol_ for calcium caseinate premixes [53]. These results indicate that 65% moisture content may realize a reasonable concentration of calcium ions during the warming process and result in stronger cross-linking. Up to a certain temperature, tan δ is generally higher during the cooling process than during the heating process (Figure 4c). Above 40 °C, tan δ increases steeply as the protein network weakens because calcium bridges are reduced and hydrophobic interactions become more dominant [56]. After 720 s of temperature-rise and 720 s of temperature-fall periods, casein–casein interactions decreased in number and strength, which resulted in a less rigid para-casein network. With this, *T*_gel–sol_ in the temperature-fall period is lower than in the temperature-rise period, and was not significantly influenced by the moisture content.

The macro-structure (Figure 3a(A1–A3)) shows the effect of moisture content on calcium caseinate solubility. The extrudate with 60% moisture content showed a transparent texture, but extrudate samples with 65% and 70% moisture content were not transparent. In this study, increasing the moisture content decreased the water-soluble protein content, increased the insoluble particles, and increased the level of phase separation, which significantly increased the fibrous degree. This is in good agreement with the textural data (Figure 2a). The thermo-rheological properties also showed a strong correlation with the bubble morphology and the flow morphology. The complex viscosity of the material decreased steeply with increasing temperature. This enabled small air bubbles to coalesce into larger ones or to escape from the material. The flowability of material in the cooling die is an essential precondition for the formation of shear and elongational flows [57]. During the temperature-fall period, the *T*_gel–sol_ of the material was approximately 50 °C. The material was cooled rapidly from the viscous state to the rubbery state with the cooling die unit at 30 °C. This rapidly reduced the flowability of materials. When the cooling die temperature was set to 45 °C (i.e., slightly less than *T*_gel–sol_), the material slowly solidified from the outer layer to the core. The material in the core was in the viscoelastic flow state, which ultimately generated an elongational flow (Figure A1a,d). This may explain why the material formed a better fibrous structure when the temperature of cooling die units was set to 45-45-30 °C (Figure 3d(A1)).

## 4. Conclusions

Increasing the moisture content, the extrusion temperature, and the cooling die temperature reduced the hardness and chewiness of the extrudate, while increasing the fibrous degree. The screw speed had a limited effect on textural properties and fibrous appearance. Causing the coalescence and escape of air bubbles, the rapid decrease in viscosity of the material at high extrusion temperatures reduced the fibrous degree. Due to high moisture content or high cooling die unit temperature, the viscosity of calcium caseinate decreased, which resulted in a pronounced fibrous structure with a further elongated V-shape pattern. According to a comparison of the calcium caseinate extrudate with the real aquatic product, further improvement is required, not only for the fibrous degree and textural properties at the macro-scale, but more importantly, for the fibrous morphology at the micro-scale.

## Figures and Tables

**Table 1:** Extrusion parameters during calcium caseinate extrusion trials.

Moisture Content (%)	Extrusion Temperature (°C)	Screw Speed (rpm)	Cooling Die Unit Temperature (°C)
60	50	50	45-45-30
65	50	50	45-45-30
70	50	50	45-45-30
70	50	50	45-45-30
70	70	50	45-45-30
70	90	50	45-45-30
70	50	50	45-45-30
70	50	80	45-45-30
70	50	110	45-45-30
70	50	50	45-45-30
70	50	50	45-30-30
70	50	50	30-30-30

**Table 2:** Texture profile analysis (TPA) of extrudates under different processing conditions and three aquatic products ^1^.

Variable Parameters		Hardness (g)	Adhesiveness (g·s)	Springiness (g)	Cohesiveness	Chewiness
Moisture Content (%)	60	1106.46 ± 238.21 ^a^	16.46 ± 7.17 ^b^	0.877 ± 0.079 ^a^	0.742 ± 0.019 ^a^	726.37 ± 207.24 ^a^
	65	767.26 ± 25.16 ^b^	25.34 ± 2.62 ^ab^	0.903 ± 0.087 ^a^	0.738 ± 0.007 ^a^	510.09 ± 37.31 ^ab^
	70	533.88 ± 15.72 ^b^	28.21 ± 1.61 ^a^	0.926 ± 0.013 ^a^	0.709 ± 0.004 ^b^	350.54 ± 11.64 ^b^
Extrusion Temperature (°C)	50	533.88 ± 15.72 ^a^	28.21 ± 1.61 ^a^	0.926 ± 0.013 ^a^	0.709 ± 0.004 ^b^	350.54 ± 11.64 ^a^
	70	392.02 ± 51.05 ^b^	22.19 ± 8.71 ^a^	0.768 ± 0.012 ^b^	0.740 ± 0.009 ^a^	223.40 ± 33.64 ^b^
	90	377.84 ± 39.91 ^b^	27.86 ± 5.47 ^a^	0.773 ± 0.014 ^b^	0.746 ± 0.015 ^a^	217.71 ± 22.49 ^b^
Screw Speed (rpm)	50	533.88 ± 15.72 ^b^	28.21 ± 1.61 ^b^	0.926 ± 0.013 ^a^	0.709 ± 0.004 ^a^	350.54 ± 11.64 ^b^
	80	714.39 ± 46.02 ^a^	49.22 ± 16.98 ^a^	0.907 ± 0.031 ^a^	0.708 ± 0.029 ^a^	459.17 ± 51.84 ^a^
	110	719.63 ± 80.33 ^a^	36.76 ± 5.01 ^ab^	0.917 ± 0.038 ^a^	0.714 ± 0.023 ^a^	473.18 ± 79.10 ^a^
Cooling Die Unit Temperature (°C)	45-45-30	533.88 ± 15.72 ^b^	28.20 ± 1.61 ^b^	0.926 ± 0.013 ^a^	0.709 ± 0.004 ^b^	350.54 ± 11.64 ^b^
	45-30-30	578.99 ± 39.06 ^b^	36.83 ± 4.10 ^a^	0.831 ± 0.027 ^b^	0.779 ± 0.013 ^a^	374.15 ± 20.27 ^b^
	30-30-30	733.01 ± 58.65 ^a^	43.21 ± 5.60 ^a^	0.790 ± 0.016 ^c^	0.773 ± 0.008 ^a^	448.30 ± 42.73 ^a^
Aquatic Product	Cod	1099.70 ± 118.92	6.57 ± 4.33	0.627 ± 0.008	0.502 ± 0.094	340.74 ± 35.65
	Scallop	268.36 ± 26.00	7.53 ± 1.21	0.688 ± 0.038	0.316 ± 0.009	58.31 ± 6.71
	Salmon	638.40 ± 27.03	11.51 ± 5.51	0.666 ± 0.051	0.364 ± 0.080	155.56 ± 39.68

^1^ All values shown are mean ± SD (*n* = 3). Means with different lowercase letters within columns for each parameter are significantly different (Least Significant Difference test, *p* < 0.05).

**Table 3:** Gel–sol transition temperature (*T*_gel–sol_) of calcium caseinate premixes at the moisture content of 60%, 65%, and 70% during temperature sweep experiments.

Moisture Content (%)	Temperature-Rise Period	Temperature-Fall Period
	*T*_gel–sol_ (°C)	*T*_gel–sol_ (°C)
70	66.7	51.1
65	83.4	49.3
60	61.3	49.5

## Data Availability

The data presented in this study are available on request from the corresponding author.
